# Uncontrolled hypertension in Ethiopia: a systematic review and meta-analysis of institution-based observational studies

**DOI:** 10.1186/s12872-020-01414-3

**Published:** 2020-03-11

**Authors:** Firehiwot Amare, Bisrat Hagos, Mekonnen Sisay, Bereket Molla

**Affiliations:** 1 Clinical Pharmacy Unit, School of Pharmacy, College of Health and Medical Sciences, Haramaya University, Harar, Ethiopia; 2 Social Pharmacy Unit, School of Pharmacy, College of Health and Medical Sciences, Haramaya University, Harar, Ethiopia; 3 Pharmacology Unit, School of Pharmacy, College of Health and Medical Sciences, Haramaya University, Harar, Ethiopia

**Keywords:** Hypertension, Cardiovascular disease, Ethiopia

## Abstract

**Background:**

Uncontrolled hypertension is one of the major risk factors of cardiovascular and cerebrovascular diseases. The prevalence of hypertension in Ethiopia is expected to reach up to 30%. The aim of this study was to determine the prevalence of uncontrolled hypertension among hypertensive patients on treatment in Ethiopia.

**Methods:**

Electronic databases and search engines including EMBASE (Ovid), PubMed/Medline, and Google Scholar were searched for original records in the English language addressing hypertension control in Ethiopia from 2000 to 2018. Data were extracted using a format prepared in Microsoft Excel and exported to STATA 15.0 software for analyses. The study protocol is registered at PROSPERO with reference number ID: CRD42018116336.

**Results:**

A total of 13 studies with 5226 hypertension patients were included for systematic review and meta-analysis. The pooled prevalence of uncontrolled hypertension in Ethiopia was 48% (95% confidence interval (CI): 36, 61%). The result of the sub-group analysis, based on the year of publications, revealed that the prevalence of uncontrolled BP was highest in 2016 (63%; CI: 60, 67%) and in 2015 (59%; CI: 53, 65%). Univariate meta-regression revealed that sampling distribution was not a source of heterogeneity for the pooled estimate as well as the sub group analysis.

**Conclusion:**

The prevalence of uncontrolled hypertension was high in Ethiopia. This alarming public health issue fuels the ever-increasing cardiovascular and cerebrovascular diseases. The ministry of health has to design a policy and implementation mechanisms to reduce uncontrolled hypertension prevalence and improve awareness on blood pressure control.

## Background

Hypertension is the major contributor to global burden of cardiovascular morbidity and mortality [1]. Currently, more than 1.4 billion of the world’s population have hypertension [2] and this figure is expected to rise to 1.6 billion by the year 2025 [3]. The cardiovascular and cerebrovascular complications of hypertension are the most important causes of non-communicable diseases (NCD) related morbidities and mortalities [4]. As hypertension is a preventable risk factor, collaborated actions can prevent the development of complications [5].

Meta-analysis of observational studies in Ethiopia estimated the prevalence of hypertension to be between 20 and 30% [6, 7]. According to WHO, 39% of all deaths in Ethiopia are due to NCDs of which 16% is attributed to cardiovascular diseases (CVD) [8]. Uncontrolled hypertension is one of the major causes of heart failure, chronic renal failure, and ischemic and hemorrhagic strokes which impose severe financial and service burdens on health systems [9, 10]. The control of hypertension within a target goal of blood pressure (BP) plays a critical role in reducing associated CVD. However, hypertension remains inadequately controlled in clinical practice [11, 12]. This would increase the burden of CVD on the health system. The proportions of patients treated for hypertension with uncontrolled BP reported across the country vary substantially. However, these data have not been meta-analyzed to provide pooled estimate of the prevalence of uncontrolled BP among treated hypertensive patients. Therefore, the aim of this study is to examine the prevalence of uncontrolled BP among treated hypertensive patients in Ethiopia. Determining the prevalence will help to comprehend the magnitude of the problem and develop strategies to reduce the imposed burden of CVD.

## Methods

### Study protocol

The Preferred Reporting Items for Systematic reviews and Meta-analyses (PRISMA) was used in the identification of records, screening of titles and abstracts accompanied by evaluation of eligibility of full texts for final inclusion [13]. The study protocol is registered at PROSPERO with reference number ID: CRD42018116336 and the published methodology is available from: http://www.crd.york.ac.uk/PROSPERO/display_record.php?ID=CRD42018116336.

### Data sources and search strategy

Literature search was done from PubMed/Medline, EMBASE (Ovid® interface) and Google Scholar. Advanced search strategies were used to retrieve relevant findings, by restricting the search for studies on human and published in English. HINARI interface was used to access articles published in subscription based journals and indexed in Science-Direct and Wiley online library. Gray literatures from organizations and online university repositories were accessed through Google Scholar. Key words and indexing terms were used to retrieve articles that were published from 2000 onwards. The key words used for searching were “hypertension”, “high blood pressure” [MeSH] and “Ethiopia”. Boolean operators (AND, OR) were also used in the identification of records. The search was conducted from February 1 to 14, 2019 and all published and unpublished articles available online from January 1, 2000 till the day of data collection were considered.

### Screening and eligibility of studies

ENDNOTE reference manager software version 9.2 (Thomson Reuters, Stamford, CT, USA) was used. With the help of the reference manager, duplicate records were identified, recorded and removed. Due to variation in reference styles from different sources, some references were managed manually. Thereafter, two authors (FA and BH) independently screened the titles and abstracts with predefined inclusion criteria. Two authors (MS and BM) independently collected full texts and evaluated the eligibility of them for final inclusion. In each case, the third author played a critical role in solving discrepancies that arose between two authors and in coming to a final consensus.

### Inclusion and exclusion criteria

Predefined inclusion-exclusion criteria were used to screen titles and abstracts; and evaluate full texts for eligibility. Observational studies addressing hypertension control among treated adult hypertensive patients in Ethiopia were included. Literatures published from 2000 onwards in the English language were considered. Articles with irretrievable full texts (after requesting full texts from the corresponding authors via email and/or ResearchGate), records with unrelated outcome measures, articles with missing or insufficient outcomes were excluded.

### Data extraction

Data abstraction format was prepared in Microsoft Excel. Two authors (FA and BH) independently extracted data related to study characteristics (study area, first author, and year of publication, study design, population characteristics, and sample size) and outcome of interest (hypertension control).

### Quality assessment of studies

The internal and external validity of included studies was assessed by using the Johanna Briggs institute (JBI) critical appraisal checklist for studies reporting prevalence data. Based on the checklist, the studies were graded out of 9 points (Table 1). Scores of the two authors (MS and BM) in consultation with third author (FA) (in case of disagreement between the two authors’ appraisal result) were taken for final decision. Studies with the number of positive responses (yes) greater than half of the number of checklists (i.e., score of five and above) were included in the systematic review and meta-analysis.

**Table 1:** Quality assessment of studies using JBI’s critical appraisal tools designed for prevalence studies

Study	JBI’s critical appraisal questions											
	Sample size	Q1	Q2	Q3	Q4	Q5	Q6	Q7	Q8	Q9	Score	Overall Appraisal
Lichisa et al	160	Y	Y	N	Y	Y	Y	Y	Y	Y	8	Include
Woldu et al	288	N	Y	Y	Y	Y	Y	Y	Y	Y	8	Include
Tesfaye et al	291	Y	Y	N	Y	Y	Y	Y	Y	Y	8	Include
Asgedom et al	286	Y	Y	Y	Y	Y	Y	Y	Y	Y	9	Include
Amare et al	616	Y	Y	Y	Y	Y	Y	Y	Y	Y	9	Include
Abdu et al	310	Y	Y	U	Y	Y	Y	Y	Y	Y	7	Include
Abegaz et al	561	Y	Y	Y	Y	Y	Y	Y	Y	Y	9	Include
Berhe et al	897	Y	Y	Y	Y	Y	Y	Y	Y	Y	9	Include
Muleta et al	131	N	Y	U	Y	Y	Y	Y	Y	Y	7	Include
Abegaz et al	543	Y	Y	Y	Y	Y	Y	Y	Y	Y	9	Include
Animut et al	395	Y	Y	Y	Y	Y	Y	Y	Y	Y	9	Include
Teshome et al	392	Y	Y	Y	Y	Y	Y	Y	Y	Y	9	Include
Yazie et al	356	Y	Y	Y	Y	Y	Y	Y	Y	Y	9	Include

*Y* Yes, *N* No, *U* Unclear, *Q* Question. Overall score is calculated by counting the number of Ys in each row

Q1 = Was the sample frame appropriate to address the target population? Q2 = Were study participants sampled in an appropriate way? Q3 = Was the sample size adequate? Q4 = Were the study subjects and the setting described in detail? Q5 = Was the data analysis conducted with sufficient coverage of the identified sample? Q6 = Were valid methods used for the identification of the condition? Q7 = Was the condition measured in a standard, reliable way for all participants? Q8 = Was there appropriate statistical analysis? Q9 = Was the response rate adequate, and if not, was the low response rate managed appropriately?

### Outcome measurements

The primary outcome measure in this meta-analysis is the prevalence of uncontrolled hypertension in Ethiopia. It is aimed to assess the pooled estimates of uncontrolled hypertension among treated hypertensive patients in the country. The sample size was intentionally adjusted to response rates in individual study to reduce bias in calculating the overall prevalence.

### Data processing and statistical analysis

A format prepared in Microsoft Excel was used to extract data from the included studies. The data was then exported to STATA software, version 15.0 for analyses. The percentage of variance attributable to study heterogeneity was assessed using I^2^ statistics. To ascertain variation in true effect sizes across population, Der Simonian and Laird’s random effects model was applied at 95% confidence level. The event rate (proportion) was calculated out of 1 and standard error of Logit event rate was also added with the help of Comprehensive Meta-analysis (CMA) (Biostat, Englewood, New Jersey, USA) version-3 software. CMA was also used for publication bias assessment by using the Begg and Rank correlation as well as Egger’s regression tests. Funnel plots of standard error and precision with Logit event rate was used to present the publication bias assessment. A *p*-value less than 0.05 (one tailed) was used to declare significance.

## Results

**Fig. 1:**
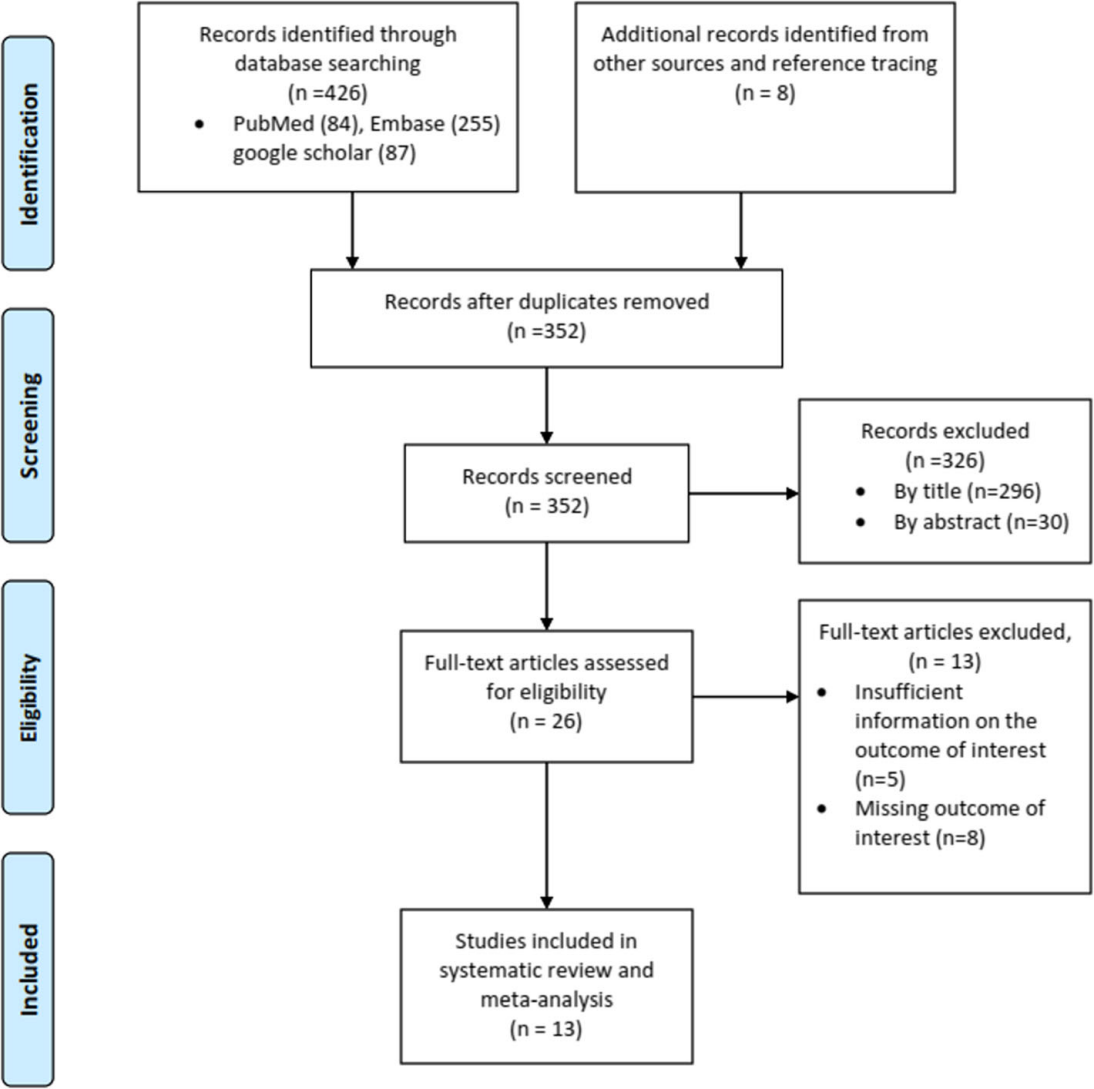
A PRISMA flowchart showing the selection process of the articles

A total of 426 studies were identified through the search of electronic databases including PubMed/Medline, EMBASE, and Google Scholar. Eight other articles were identified through reference tracing and other sources. After removing 82 duplicates through ENDNOTE reference manager and manual tracing, a total of 352 records were screened using their titles and abstracts. Then, full text assessment of 26 potentially relevant articles resulted in 13 studies that passed the eligibility criteria and quality assessment and hence included in the systematic review and meta-analysis (Fig. 1).

### Study characteristics

A total of 13 studies with 5226 hypertensive patients were included in this systematic review and meta-analysis. Among the included hypertensive patients, 2534 were found to have uncontrolled BP. Eleven of the included studies used cross-sectional study design [14–24] while the remaining two were cohort in design [25, 26]. Almost all the included studies were hospital based except for one which was conducted at health centers [18]. The year of publication of included studies ranged from 2014 to 2018. The prevalence of uncontrolled hypertension ranged from 11.42% [22] in Gondar university hospital to 69.94% in Zewditu memorial hospital, Addis Ababa [24] (Table 2).

**Table 2:** Descriptive summary of studies on hypertension control in Ethiopia included for systematic review and meta-analysis

Author	Publication year	Study design	Study population	Study area	Sample size	Event
Lichisa et al [14]	2014	CS	Hypertensive patients	Adama Hospital Medical College	160	90
Woldu et al [15]	2014	CS	Hypertensive patients	Bishoftu general hospital	288	56
Tesfaye et al [16]	2015	CS	Hypertensive patients	Tikur Anbessa Specialized Hospital	291	172
Asgedom et al [17]	2016	CS	Hypertensive patients	Jimma University Specialized hospital	286	142
Amare et al [18]	2016	CS	Hypertensive patients	Health centers of Addis Ababa	616	425
Abdu et al [19]	2017	CS	Hypertensive patients	Gondar university hospital	310	115
Abegaz et al [20]	2017	CS	Hypertensive patients	Gondar university hospital	561	167
Berhe et al [25]	2017	Retrospective cohort	Hypertensive patients	Six public hospitals in Ethiopia	897	562
Muleta et al [21]	2017	CS	Diabetic hypertensive patients	Jimma University Medical Center	131	74
Abegaz et al [22]	2018	CS	Hypertensive patients	Gondar university hospital	543	62
Animut et al [26]	2018	Retrospective follow up	Hypertensive patients	Gondar university hospital	395	196
Teshome et al [23]	2018	CS	Hypertensive patients	Debre Tabor district hospital	392	224
Yazie et al [24]	2018	CS	Hypertensive patients	Zewditu memorial hospital	356	249

*CS* Cross sectional

### Study outcome measures

#### Primary outcomes

**Fig. 2:**
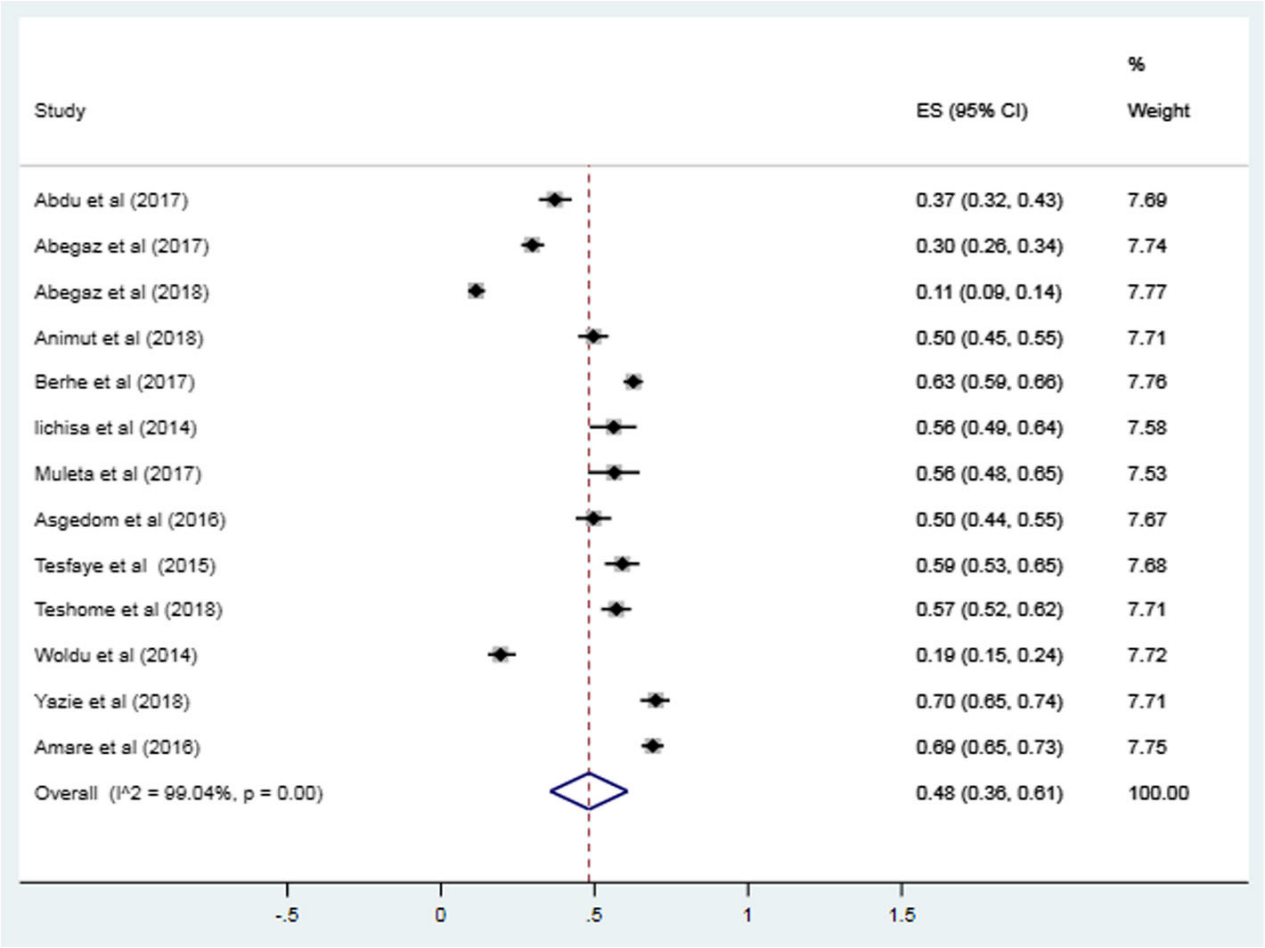
Forest plot depicting the pooled estimate of the prevalence of uncontrolled hypertension among treated hypertensive patients in Ethiopia

**Fig. 3:**
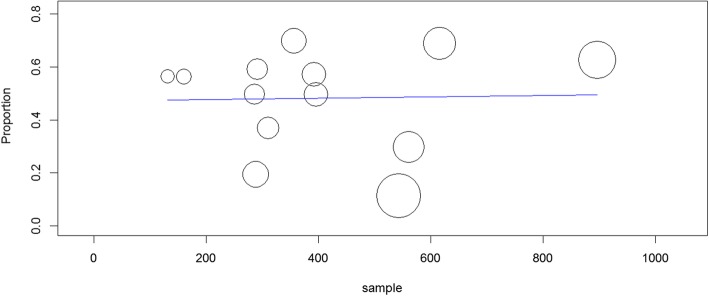
Univariate meta-regression model using sample size

The pooled prevalence of uncontrolled hypertension in Ethiopia from the 13 studies describing control of BP among treated hypertensive patients was 48% (95% confidence interval [CI]: 36, 61%). When random effects model was assumed for this meta-analysis, a high degree of heterogeneity was observed across studies as evidenced by the I^2^ statistics (I^2^ = 99.01%, *P* < 0.001) (Fig. 2). Univariate meta-regression model showed that sampling distribution is not a source of heterogeneity (regression coefficient = 0.000, *p*-value =0.92) (Fig. 3).

#### Sensitivity and subgroup analyses

**Fig. 4:**
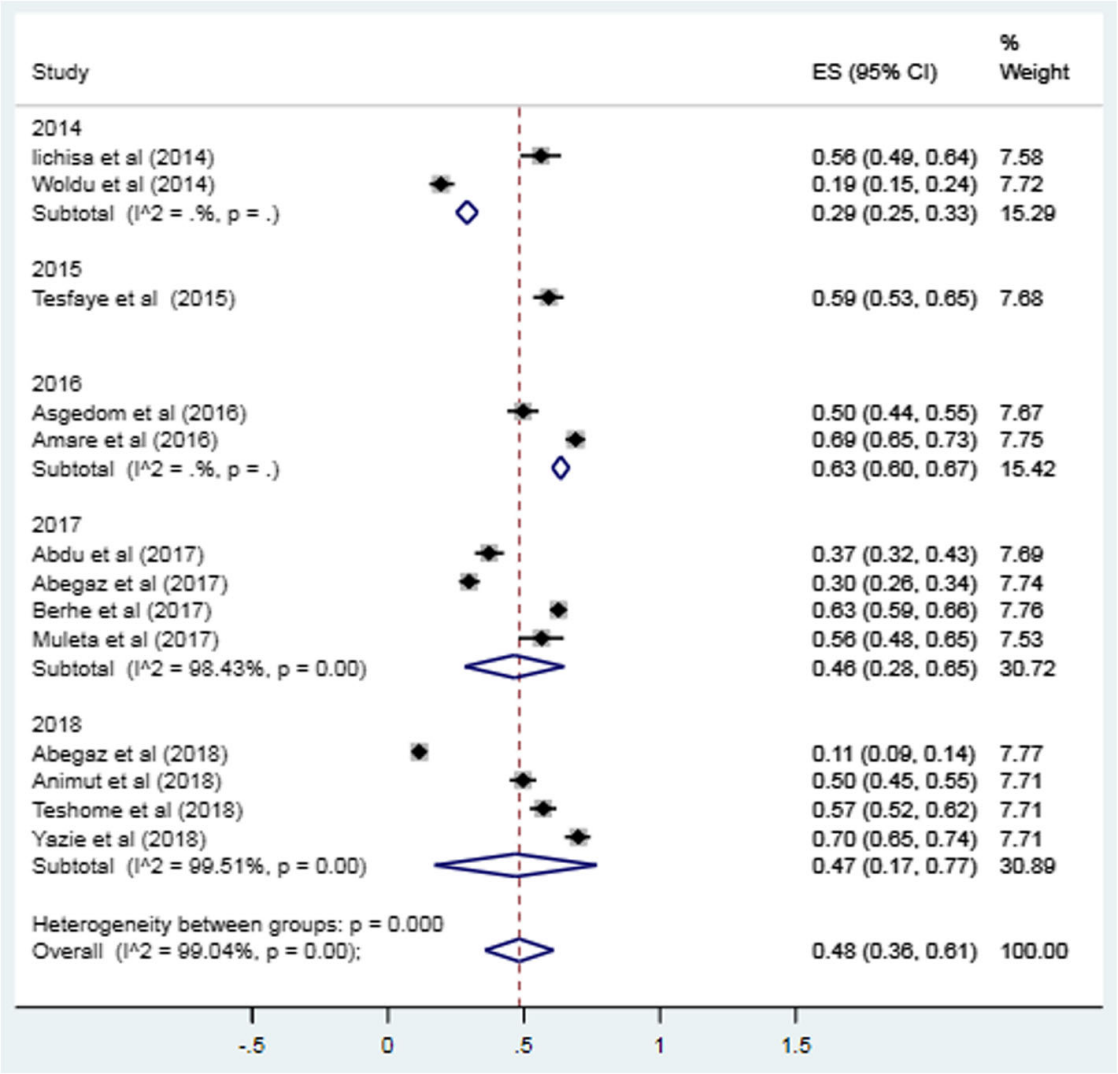
Sub-group analysis based on publication year of studies

**Fig. 5:**
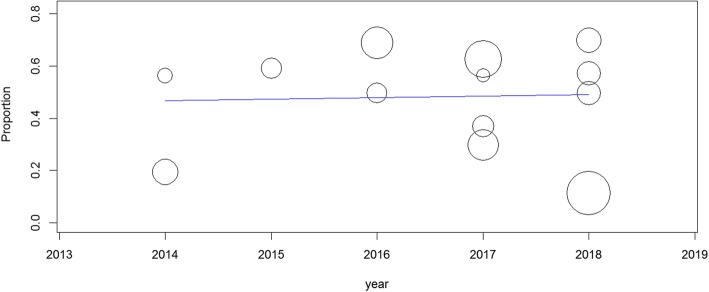
Univariate meta-regression model using publication year of studies

Sensitivity analysis was conducted by excluding outliers from the analysis. However, there was no significant change on the degree of heterogeneity even when outliers were excluded from the analysis. Therefore, all the studies that passed the quality assessment were included for the meta-analysis. A subgroup analysis was conducted based on the year of publication of the studies. The result of the subgroup analysis revealed that the prevalence of uncontrolled hypertension was highest in 2016 (63%; CI: 60, 67%) followed by 2015 (59%; CI: 53, 65%) (Fig.4). Univariate meta-regression revealed that year of publication is also not a source of heterogeneity (regression coefficient = 0.005, *p*-value =0.88) (Fig. 5).

#### Publication bias

**Fig. 6:**
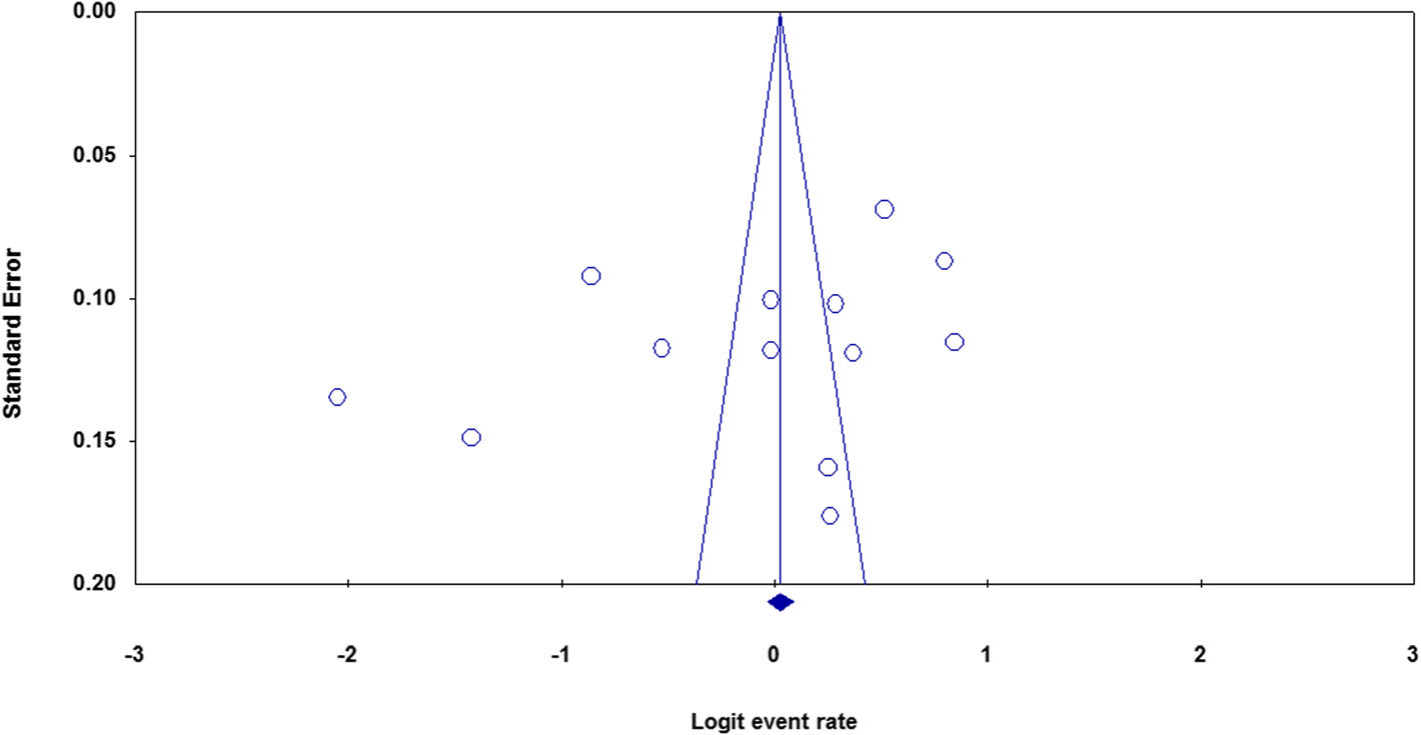
Funnel plot illustrating publication bias of included studies with Logit event rate and standard error

Publication bias was assessed by using funnel plots of standard error with logit effect size (event rate). The analysis showed that there is no evidence of publication bias on the included studies. This is confirmed by Egger’s regression test (one-tailed), *p* = 0.09 and Begg’s correlation test (one tailed), *p* = 0.15 (Fig. 6).

## Discussion

A total of 13 institution based studies with 5226 hypertensive patients were included in this systematic review and meta-analysis. In the current study, the pooled prevalence of uncontrolled hypertension among hypertensive patients on treatment in Ethiopia was 48% (CI: 36, 61%). This finding indicated that almost half of hypertensive patients who were following their treatment in health institutions (hospitals and health centers) in Ethiopia did not achieve a target BP, proven to reduce CVD risk associated with hypertension. The prevalence of uncontrolled hypertension in this study is close to the proposed WHO target control rate [1]. The result of the subgroup analysis showed the increment in the prevalence of uncontrolled hypertension from 2014 to 2017. This clearly shows the quality of health service provided for patients with hypertension. Additionally, the national burden of cardiovascular and cerebrovascular diseases, chronic renal failure and the associated morbidity and mortality are expected to rise with the uncontrolled BP [27]. Moreover, a study has shown that treated hypertensive patients but not having control were at increased risk of all cause, CVD specific, heart disease-specific or cerebrovascular disease specific mortality [28]. According to the report by WHO, only 12% of high risk persons were receiving drug therapy and counseling to prevent heart attacks and strokes [8].

The prevalence of uncontrolled hypertension in this study 48% (CI: 36, 61%) was lower than what was reported from a meta-analysis of 135 population based studies from 90 countries across the world (62.9%) and the prevalence in low- and middle income countries (73.7%) [29]. Similarly, the current prevalence was lower than a report from India (rural 89.7% and urban 79.8%) [30]; a national survey in China (91.9%) [31] and a meta-analysis of studies from Brazil [men (68.2%) and women (43.1%)] [32]. This difference might have resulted as the studies included in this meta-analysis were only institution based where there is strict control in the measurement of BP and management of hypertension.

On the other hand, the prevalence of uncontrolled hypertension in this study is in trajectory with a Kenyan national survey (48.3%) [33] and lower than studies from Dutch (30%) [34], England (23.9%), Canada (14%) and USA (21.2%) [27]. The high prevalence of uncontrolled hypertension observed in this study might have resulted from socioeconomic factors; low educational status and poverty [35]. Additionally, unavailability of or interrupted supply of medicines could have contributed to the high prevalence. As WHO stated, only 1 in 10 essential NCD medicines are reported to be available at health facilities of the country [8].

In the sub-group analysis, uncontrolled hypertension increased over the years. This is in contrary to a study that described the 25 years trend of hypertension control in India that showed a decrease from 81 to 51% [36]. Given the developing nature of the country and the burden of communicable diseases, the increase in uncontrolled blood pressure should be alarming. In order to decrease the burden of CVD associated with uncontrolled hypertension, home BP monitoring [37] and a holistic approach of patient care including pharmacists to manage patients drug therapy should be used [38].

## Conclusion

The prevalence of uncontrolled hypertension was high in Ethiopia. This is alarming as uncontrolled hypertension is associated with an increased risk of cardiovascular complications. This would impose additional burden on the health care system of the country, which is struggling to contain communicable diseases. The prevalence of uncontrolled hypertension is increasing over the years. This evidence suggests that double burden diseases are increasingly affecting Ethiopia. In light of this evidence, policy makers and health care professionals working in the area should implement interventional strategies focusing on achieving an optimal BP among treated hypertensive patients.

### Limitation of the study

The study has extensively addressed all relevant data regarding hypertension control among treated hypertensive patients in Ethiopia. However, there are certain limitations to mention. The studies included for the meta-analysis used different cut-off point to define control of BP as there was change in guideline recommendation regarding optimal BP. Additionally, the number of BP measurement used to define uncontrolled hypertension across the included studies was inconsistent.

## Data Availability

All data used for the systematic review and meta-analysis is contained within the manuscript.

## Abbreviations

BP: Blood pressure
CS: Cross sectional
CVD: Cardiovascular disease
NCD: Non communicable diseases
WHO: World health organization
